# Gingival Biopsy to Detect Mosaicism in Overgrowth Syndromes: Report of Two Cases of Megalencephaly-Capillary Malformation Syndrome with Periodontal Anomalies

**DOI:** 10.1155/2020/8826945

**Published:** 2020-09-12

**Authors:** Mathieu Marty, Carole Bonnaud, Natalie Jones, Michel Longy, Frédéric Vaysse, Eric Bieth, Isabelle Bailleul-Forestier

**Affiliations:** ^1^Paul Sabatier Toulouse III University, Department of Paediatric Dentistry, CHU Toulouse, F-31000, France; ^2^Cancer Genetics Unit & INSERM U1218, Institut Bergonié, Bordeaux University, Bordeaux, France; ^3^Paul Sabatier Toulouse III University, Genetics Unit, CHU Toulouse, F-3100, France; ^4^KU Leuven University, Belgium

## Abstract

**Background:**

Megalencephaly-capillary malformation (MCAP) is a rare overgrowth syndrome caused by postzygotic activating mutations in the *PIK3CA* gene.

**Aim:**

To illustrate the benefits of gingival biopsy in the genetic diagnosis of overgrowth syndromes.

**Design:**

Gingival biopsy was performed on a 13-year-old patient and a 16-year-old patient with MCAP and who suffered from periodontal disease. *PIK3CA* sequencing was performed on DNA extracted from gingival biopsies, blood, and saliva.

**Results:**

Pathogenic p.Glu365Lys and p.Glu545Asp *PIK3CA* mutations were found in the gingival biopsies with an allelic frequency of 22% and 35%, respectively, while they were undetectable in blood or saliva. The genetic diagnosis of MCAP through detection of *PIK3CA* somatic mosaicism in a periodontal biopsy is unprecedented.

**Conclusions:**

Considering the tissue distribution and level of somatic mosaicism for *PIK3CA* mutation, the composite embryologic origin of periodontium and its high fibroblast cell content make it an ideal target for molecular analysis in overgrowth syndromes, and multidisciplinary approach including paediatric dentists should be encouraged. In addition, our clinical findings suggest that periodontal disease is part of the MCAP phenotypic spectrum and should be systematically investigated.

## 1. Introduction

Megalencephaly-capillary malformation (MCAP) syndrome is a rare congenital condition belonging to the segmental overgrowth disorder group caused by somatic mutations in the RTK/PI3K/AKT/mTOR growth pathway [1]. In this group, the *PI3KCA*-related overgrowth spectrum (PROS) includes MCAP syndrome and several other disease entities with clinical overlap (reviewed by Keppler-Noreuil et al., 2014). MCAP syndrome (MIM#602501) is characterized by megalencephaly, capillary malformations, asymmetric growth, polymicrogyria, polydactyly, and syndactyly [2].

The *PIK3CA* gene (locus 3q26.32) encodes the 110 kD (p110) alpha subunit of the phosphatidylinositol 4,5-bisphosphate 3-kinase (PI3K) involved in intracellular signalling. The PI3K signalling pathway is important for many cellular activities including cell growth, division, migration, angiogenesis, and survival. These functions make PI3K an essential protein in the development of tissues, including brain tissue and blood vessels. Some *PIK3CA* mutations have oncogenic potential (MIM∗171834). In MCAP syndrome, *PIK3CA* mutations lead to an increased p110 alpha subunit activity that results in constitutive PI3K signalling [1].

Clinically, MCAP syndrome may result in a disproportionate head due to an enlarged brain and capillary malformations (vascular abnormalities) on the skin of the midline face, trunk, and limbs. The oral manifestations of this syndrome are not yet described; the following two case reports provide a description of the particular aspect of periodontal disease in children with MCAP.

## 2. Case Series

### 2.1. Case Report 1

To illustrate the interest of gingival biopsy in the diagnosis of such syndrome, we report here the cases of a 13-year-old patient who consulted in Toulouse University Hospital in March 2015 for bleeding gums. Her medical history revealed a clinical diagnosis of MCAP not confirmed by genetics.

Her medical drug therapy consisted of analgesics for chronic pain in the lower right limb with lymphatic malformations. Lymphatic drainage and the wearing of a compression garment at night were implemented. She also had a slight inequality in the length of her lower limbs (2-3 mm), for which orthopaedic soles were made.

During early childhood, the lymphatic malformations of the right hemiface resulted in bites on the internal surface of the overgrown cheek. A lipoaspiration was thus carried out in 2006 followed by wearing of a contention mask for 1 month in order to decrease the volume of the right cheek. The Institutional Review Board approved this study, and the treatment was performed after obtaining written consent from the parents.

**Figure 1:**
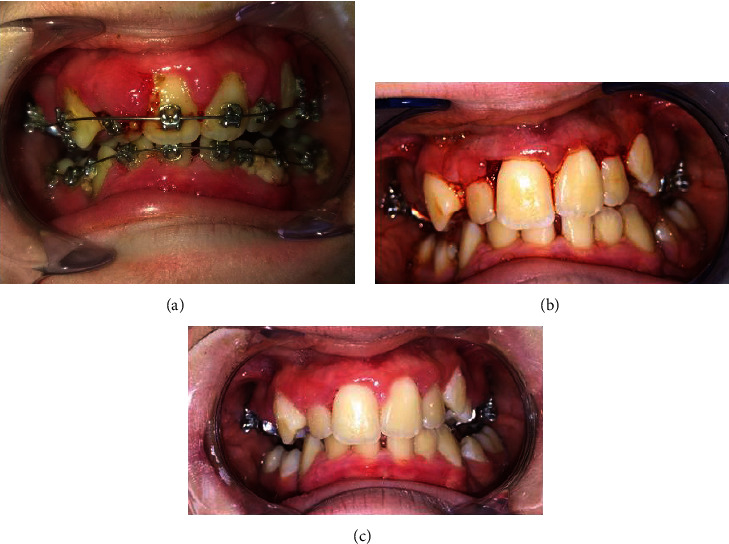
(a) Intraoral initial situation. (b) Intraoral situation after root planning and gingivectomy. (c) At 6 months, end of the first phase of periodontal treatment.

Exobuccal examination revealed slight right side dominant facial asymmetry. We did not observe cutaneous capillary malformations on her face. The endobuccal clinical examination (Figure 1(a)) revealed the presence of a bimaxillary multiring orthodontic appliance and major, generalized gingival inflammation with spontaneous bleeding. Gums and papillae were oedematous and erythematous. The mesial papilla of the right upper lateral incisor was the most affected and had a budding appearance. Oral hygiene was poor, and plaque deposits were present on all dental surfaces (which may have been partly explained by the difficulty of brushing and the retention of plaque caused by the orthodontic appliance and also by the pain upon contact with inflamed gums).

Classical periodontal therapy was established, including the following:

- Explanations to the patient and her parents about the illness (causes, consequences, and treatments)
- Oral hygiene motivation and education
- Antibiotic treatment (amoxicillin and metronidazole)
- Removal of orthodontic appliance
- Tooth scaling

After reevaluation, root planing was performed in two sessions under conscious sedation with nitrous oxide (due to the age and anxiety of the patient).

A gingivectomy was also performed, and samples of periodontal tissue in the most affected area (mesial papilla of the right upper lateral incisor) and saliva were sent for genetic analysis (Figure 1(b)).

After 6 months, the clinical improvement was notable but gingival inflammation persisted, with induced bleeding and a budding and erythematous appearance of certain papillae, especially in the mesial papilla of the right upper lateral incisor (Figure 1(c)).

A literature search was conducted for intraoral periodontal and mucosa abnormalities associated with MCAP syndrome. No articles reporting intraoral lesions in MCAP subjects were found. However, capillary malformations of the face were reported [3, 4].

Gingival biopsy was performed on the mesial papilla of the right upper lateral incisor which was the most budding and erythematous area. Genetic analysis was carried out on the *PIK3CA* gene by a next-generation sequencing process with confirmation of the variants by the Sanger method [5].

**Figure 2:**
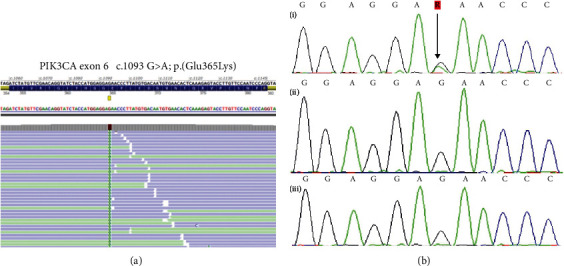
Identification of a mosaic PIK3CA missense mutation in a gingival biopsy. (a) Next-generation sequencing (NGS) of the gingival biopsy sample identified a missense mutation in exon 6 of the PIK3CA gene, as indicated by the yellow square. Twenty-two percent of reads showed the G>A change (324/1475 reads, 174+ 150-), suggesting a potential mosaic mutation. (b) Direct Sanger sequencing of 3 independent samples from the patient confirmed the presence of the missense mutation in the (i) gingival biopsy sample and showed the absence of this mutation in the (ii) blood and (iii) saliva samples, supporting the hypothesis of a mosaic mutation.

The *PIK3CA* gene analysis was performed on DNA from gingival biopsy, blood, and saliva using next-generation sequencing (NGS) with confirmation by the Sanger method. The pathogenic mutation NM_006218.2:c1093G>A (p.Glu365Lys) was found only in the gingival biopsy with an allelic ratio of 22% (Figure 2).

This missense mutation was previously reported with the same level of mosaicism in skin-derived fibroblasts of a MCAP patient [6].

### 2.2. Case Report 2

**Figure 3:**
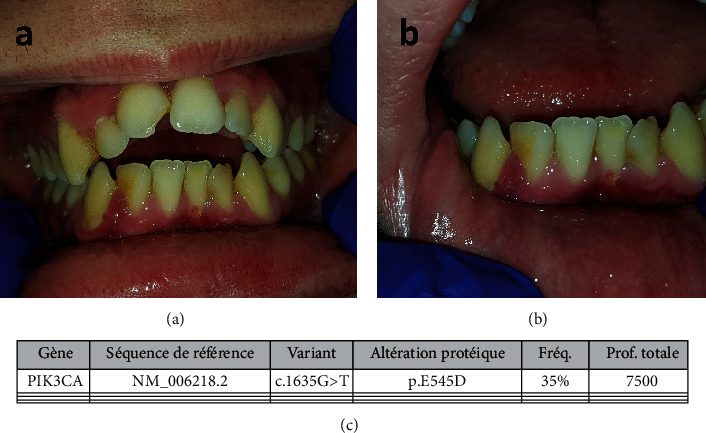
(a) Intraoral situation for patient 2. (b) Aspect of the inflammatory papillae between teeth 42 and 43. (c) Result of the genetic analysis.

The second patient was a 16-year-old boy, who came to Toulouse University Hospital for gingival bleeding in January 2018. The molecular diagnosis of MCAP was previously made on a brain tissue sample. The medical history revealed a cerebral surgery in 2013. The clinical oral examination revealed a gingival inflammation, with induced bleeding and the same erythematous appearance of certain papillae (Figure 3(a)). Gingival biopsy was performed on the distal papilla of tooth 42 (Figure 3(b)), and a saliva sample was taken. Here again, genetic analysis was carried out on the *PIK3CA* gene by a new-generation sequencing process with confirmation of the variants by the Sanger method. On the gingival sample, a rare missense mutation was found (Figure 3(c)): NM_006218.2c.1635G>T p.E545D (p.Glu545Asp). The allelic ratio of this mutation was 35%, which reflects once again the probable existence of cellular mosaicism for this variant found in the gingival biopsy. This hypothesis is supported by the absence of detection of this variant by Sanger sequencing of saliva DNAs.

## 3. Discussion

This case series is the first description of periodontal disease linked to MCAP syndrome. The lack of information on periodontal and mucosal intraoral health could be partly explained by the young age of previous subjects, who were mostly young children and even infants. Periodontitis prevalence increases with age, and moreover, periodontitis cannot exist without teeth. A global clinical examination often fails to consider the oral cavity because it is carried out by medical teams that, although often multidisciplinary, do not include dental surgeons.

This lack of intraoral information on the subjects of these studies contrasts with the feelings of dental surgeons who have had the opportunity to manage patients with MCAP. They notice an atypical (budding and erythematous) gingival appearance and an increased frequency of gingival and periodontal pathologies.

Additional case reports will be required to determine the set of associated periodontal and mucosal pathologies found in MCAP patients.

Concerning the first case, the identification of mosaic missense mutations in the *PIK3CA* gene in a periodontal biopsy is unprecedented. However, it is not the first time that a mosaic mutation of the *PIK3CA* gene is found in an intraoral sample. McDermott et al. identified a mosaic mutation of the *PIK3CA* gene (c2176G>A) in the DNA of a permanent tooth. Among the DNA of the blood, hair, and dental samples, it was the dental DNA that showed the highest degree of mosaicism [7]. A mosaic mutation of the *AKT1* gene, which occurs in the same intracellular signalling pathway as the *PIK2CA* gene, was identified in an odontogenic cyst of a patient with Proteus syndrome [8].

Gingival biopsy is rarely used for the diagnosis of genetic diseases unlike blood or skin samples [9]. Nevertheless, it deserves to be better known as it involves minimally invasive access and the opportunity exists to perform it in the same session as the dental care, thus taking advantage of local anaesthesia. Moreover, considering the tissue distribution and level of mosaicism in *PIK3CA* mutation, the complex embryologic origin of periodontium and its high fibroblast cell content make it an interesting target for molecular analysis in overgrowth syndromes. For the same reason, the periodontium is more interesting to detect genetic variations than buccal mucosa biopsy or saliva collection. Moreover, greater attention should be paid to hypertrophic gingival lesions in such syndromes.

In those two case reports, the dental surgeon helped in the diagnosis of MCAP. As part of the multidisciplinary care of these patients, a deeper involvement of paediatric dentists should be considered for the treatment of any associated periodontal abnormalities as well as for taking samples for genetic analysis and diagnosis.
